# Dr. Taylor on Filling Teeth

**Published:** 1851-04

**Authors:** 


THE
### DENTAL REGISTER OF THE WEST.

VOL. IV. APRIL, 1851. NO. 3.

### DR. TAYLOR ON FILLING TEETH.

*Continued from the January number.*

The fourth order of cavities in my classification are the palital, and occurs less frequently than either of the three already described. They will more frequently be found on the anterior and posterior molars above, and on such of these teeth as have well and deep marked sutures, running across from their labial to palital face ; or, as is sometimes the case, the suture dividing only the palital face, and then losing itself in the posterior depression on the grinding surface of these teeth. On the palital face, however, of the lateral incisors a depression is often met with, and then we may expect a decay presenting cavities of this order. The front incisors and canine occasionally present this condition of decay. The bicuspids scarce ever. In the teeth of the inferior maxillary they are scarce ever found. I do not now recollect of ever having met with over a dozen cavities of this classification in these teeth.

We have at times a recession of the gums from the necks of the teeth and when this is accompanied by a vitiated condition of the secretions of the mouth, we will sometimes find cavities of this order, running across the palital neck of the teeth.

This condition of decay, and that induced by clasps for artificial teeth present the most difficult of this kind to fill. These cavities, so long as they are confined to the crown of the teeth, and do not dip under the edge of the gum, are more easily managed than any other except the central, and as the operation in these is much the same it will require but little explanation. When small and situated on the line of the sutures or the depression in the incisors and canine as alluded to, they are readily prepared for the filling by the use of the burr drills and excavators. When in the sutures, however, they will often run along these, forming a long narrow cavity not very manageable with the drill. These cavities, when of considerable depth, should be of the same size at the opening of the cavity as within, and in introducing the gold, the first block or fold of gold should be placed in that part of the cavity nearest the gum. After the gold has been thoroughly compacted and made flush with the border of the cavity, the filling may be filed level with the enamel and depressed on a line with the suture, carrying out to some extent, at least, the natural form of the teeth.

The palital cavities found on the neck of the teeth present more difficulties in the operation of filling. The posterior and anterior part of these cavities will generally be found very shallow, and the more they circle the necks of the teeth the more difficulty will be experienced in the operation; sometimes requiring very much the same treatment as that described for the labial cavities on the posterior molars in the inferior maxillary. It will not do in this condition of decay to make the floor of the cavity level; for if this be the case, and the decay is on a bicuspid, or passes over the inner root of a molar, the nerve may easily be exposed. A proper knowledge of the location of the nerve will always guide us in the formation of these cavities, and indeed in all deep cavities must to a certain extent control the operation.

One difficulty almost always met with deserves a passing notice. It is, however, more a condition of things than really a part of the operation, yet such a condition as very much to increase the difficulty of forming the cavity and filling the teeth. I allude to the extreme tenderness of tooth bone most usually met with in this condition of decay, and this is especially the case when it is the esult of injury from clasps. It is evident that friction cannot alone be the cause of this difficulty. I have sometimes thought it had but little to do in the matter. We have here two characteristics of decay; one, in which the cartilaginous portion of the tooth appears to have been removed, leaving the dentine whiter than natural, and which crumbles readily under the instrument. The other is the loss of the earthy portion, leaving the cartilaginous portion soft and spongy. If now galvanic action is at work, and incites to decay or decomposition of the original elements of the tooth; why, in one month produce one state of things and in another the reverse? May not the galvanic action be changed by the condition of the fluids of the mouth? We know that one acid acts upon the lime of the tooth, and another on the cartilage, and I believe it is an admitted fact that in galvanic action heat is generated, and acids we know when heated act more powerfully than when cool. The investigation of this subject is certainly worthy the attention of the profession; and for this reason, that a dentifrice or wash which would be a proper and an efficient neutralizer of one acid is not for another; in one case it may be of decided benefit, and in the other of no use at all. Washes and dentifrices should never be prescribed for the gums and teeth without a careful investigation of the nature of the disease with which the gums or teeth may be affected. This tenderness of the teeth of which I have been speaking is often annoying to the operator and occasions much pain to the patient, and when met with I generally depend on the excavators for the preparation of the cavity. If there is no danger of exposing the nerve, I gradually work a very sharp excavator through the decay to the healthy bone, and with a firm and steady hand pass the instrument around under the carious portion. After this, I have but little further trouble in removing the decay and forming the cavity. I should want the class of cavities now under consideration slightly enlarged within, merely enough so, that the gold first introduced (commencing at the posterior part of the cavity) may not be displaced by the last fold or blocks introduced. It should be remembered that often the outer circle of these cavities is much or at least some longer than the inner, and the pressure made to introduce the last portion of the plug would be rather calculated to displace the first. The form of cavity, rather dove-tail, will, when the first blocks which have been introduced and in a measure compressed and made solid, prevent any displacement of the gold. In this condition of cavity I use almost entirely my gold rolled into blocks, made sufficiently large, so that each block as introduced shall fill up the cavity in its breadth, so that at the insertion of each block it is forced back and down to its proper place and thus consolidated as I advance in the filling. The anterior and posterior wall of these cavities should be sufficiently deep, even if cut into the healthy bone as to afford a hold for the first and last blocks inserted. After proper compression and consolidation of the plug it should be filed smooth and rounded to the shape of the tooth, when it is ready for polishing.

The compound cavities are generally of the following kind: first, the approximal and central uniting; second, the central and labial, and sometimes the approximal and palital. Those occurring most frequently are the approximal and central, and these in the molars and bicuspids, superior and inferior. We have here a form of cavity requiring great care and skill to properly fill—that portion of the bone and enamel which separates the two cavities and which forms the upper border of the approximal has been broken away, destroying, as a matter of course, the wall which forms a part of either cavity and which is so essential for the proper retention of the plug. We have in connection with this, an extensive decay—both cavities may indeed have been large before their union. As an illustration of these cavities and the manner of filling, we will take the anterior molar of the inferior maxillary, decayed on the posterior approximal surface, and also in the center. The approximal cavity is large and has extended itself to the central. The first thing here to be done is the separation of the molars, and if the decay is as extensive as is generally found, the posterior approximal surface of the decayed molar should be cut and filed away, so that the posterior prominences shall be beveled off, forming a beveled but somewhat rounded surface from a line of union of these two cavities down to the neck of the tooth. This condition of things will at times be somewhat controlled by the depth and condition of decay.

In the formation of the cavity, care should be taken that the border circling with the gum should be well formed, and at least not diminish the size of the cavity within. But the greatest care is requisite in the formation of these cavities, at that point where the two have united. The cavity is generally most shallow at this part, and unless it fully maintains its size as it passes in, the plug will not be retained; but if the cavity at this point slightly enlarges within, no difficulty need be expected in the securing of a good and durable plug. The central portion of these cavities I form much as I should a central cavity, only changing when it unites with the approximal to suit the altered condition of the parts made necessary by the union of the two.

In the introduction of the gold I commence in the approximal part of the cavity, but I should first remark that I adjust my napkin and speculum just as if I was about to plug a central cavity. In the preparation of my gold, I take care that the first block is of sufficient size to extend from the labial to the palital wall of the cavity, and compress this to its place, being careful to fill up that portion of the cavity nearest the gum first, so that the remaining blocks shall wedge in on top of this until the cavity is completely filled to a level with the floor or bottom of the central part. I then have a few blocks long enough to extend into this, and thus fill in part the central portion in this way, leaving sufficient depth of cavity, however, to receive a central plug, which is inserted with blocks and folds of gold in the usual manner, finishing, however, at that part where the approximal and central unite, by securely wedging in a few folds of gold under the beveled wall which is here made for this purpose. The compressing instruments are now applied until the gold is completely consolidated, and the gold filed level with the margin of the cavity, using such instruments as are necessary for both the approximal and central fillings.

The plugging forceps first used in this operation are so bent that the point turns back towards the handle of the instrument and the points of the forcep blades flattened just the reverse of those used in the central cavities; these latter, are bent for the lower molars at near right angles.

We have here an approximal cavity, the upper margin of which has been destroyed, yet the plug secured in part by the central filling. All the cavities of this class found in the molars and bicuspids can generally be treated in the manner described.

The central and labial sometimes unite and are found most frequently in the molar teeth. If the central is large and deep and the labial small, and situated about midway on the crown of the tooth, the bottom of the central cavity will be below the lower wall of the labial cavity and the union is complete up to the grinding surface of the tooth. The foil here should be introduced first into the central cavity, and this should be done, compressing one or more blocks laid in on their sides, until the cavity is filled up to a level with the labial decay. Then introduce blocks on end in the posterior part of the central cavity, until it is filled up to a line with the labial; then fill up the labial letting the blocks extend through the central cavity. When this is done finish the anterior portion of the central plug; before compressing, force into the center of the central plug, which is formed by the blocks which make the labial plug, a sharp pointed plugger—make an opening as large as possible by forcing the gold against the anterior, posterior, and palital walls; then fill this with one or more small blocks, or a few folds of gold, compress the plug and file the labial filling level with the rounded face of the tooth, and trim the central as in other cases. Should the labial cavity be large and the central small, fill first the labial, and when filled up to the floor of the central, use blocks of gold which will pass into this and rest against its palital wall, and when the labial cavity has been well filled, should the central be incomplete, open in the center as before and fill solid.

In the molar teeth a central and palital cavity may unite, when the same treatment can be adopted with the same beneficial results ; or, if a portion of the palital wall of a central cavity should give way, the same treatment is admissible and affords, as I think the best possible chance for a perfect filling. One of my inferior posterior molars a few years since gave way in this manner, and after one or two ineffectual efforts to have it filled in the ordinary way, my brother, Dr. E. Taylor, plugged it in the manner which I have described, and nothing could be more satisfactory. The ends of the blocks, in part, form the palital face of the tooth, and were filed and trimmed to suit its shape.

One other condition of compound cavity is worthy of especial notice. This is palital and approximal uniting in the lateral incisors. The palital cavity is here generally near the gum, and is preceded by a natural indentation in the palital face of the tooth—a burr head drill is generally used for the preparation of these cavities for the plug, and when the approximal unite with this, it is generally near the neck of the tooth, or at what might be called the base of the approximal cavity. I first prepare my palital cavity, then adapt the approximal to suit, not enlarging any more than possible the opening which unites them. I then fill the palital ; then commence the approximal plug at the base of the cavity : next the palital filling, and finish at the point under the cutting edge of the tooth. When the fillings are compressed, trim and file to suit the natural form of the tooth.

Other specific cavities might be alluded to, yet I have already occupied, perhaps, too much time and space on this subject. True, there is none other so important to the profession, and, none having so few specific directions, by which the young operator may be guided in the performance of this difficult operation. Much might be said in relation to the position of the patient's head, and also to the position of the operator which I have not alluded to. Perhaps no two operators will always take the same position in the use of even the same instrument. That which is most important I have tried to make as plain and practicable as possible.

I have reserved till now the finishing part of the operation, which is the polishing of the plug. This has been done because about the same process is needed in all cases. The surface of the small and large fillings should alike present a fine and polished appearance. This is not only requisite for the general appearance of the plugs, but also that they may be smooth to the tongue and not be injuriously acted upon by use of tooth pick, brush, &c.

For the finishing of many small plugs, the burnisher is almost all that is necessary, using when necessary the Arkansas stone or the Scotch stone to grind off any scratches that may have been left by the file and scraping instruments and which do not yield readily to the burnishing instrument.

The file I regard as an indispensable instrument in the preparation of a plug for burnishing. These I have alluded to already, and they should be used to file off all the protruding gold and give the plug that external form which may be desirable and unless they leave the surface of the filling to some extent smooth, I should regard the gold but as poorly introduced.

There should be no ragged projecting pieces of gold around the border of the filling; to avoid this a small and somewhat blunt pointed instrument should be passed entirely around the outer border of the filling; compression made all round and the rough edges of the plug trimmed away from the margin of the cavity.

The last file used on the surface of a filling should be of the finest quality of gold files, and if they have been somewhat worn all the better.

The smoother the plug is left by the file, the less labor will itrequire to finish the operation. After the file has been used and the plug trimmed so that the antagonizing tooth does not strike first on the gold, I take a suitable burnisher and make hard compression over the entire surface of the plug; then apply the Scotch stone, (such as jeweller's use,) and grind out all the scratches left on the gold. The Arkansas stone I have found to clog up and it is not expeditious enough. When the plug is inaccessible to the use of these stones which are made of different shapes to suit almost all conditions, I take that which has been pulverized, and use this with a stick of wood, or oftener with a rounded or flattened burnisher; when this operation has been carried to sufficient extent, wipe off the plug, and with a little rouge or castile soap and burnisher, finish. In large fillings I have found it advantageous and this part of the operation much expedited by using rougher stones, those which cut faster, or for the approximal fillings, emory and glue on a tape which is passed rapidly between the teeth, and if the emory is fine it leaves a smooth surface, which is soon finished by the burnisher. Dr. Parmley's "argillaceous tooth polishers" I have often used and find them to answer a good purpose in preparing the gold for a finer material. The plug should be left perfectly smooth, free of all scratches and indentations, and reflect like a mirror the instrument as it passes over it. All should try and attain this object; true, they may arrive at it in different ways, but I think all will admit that the better the gold is introduced and consolidated, the easier will this be accomplished.

The subject of destroying the nerve and filling thereafter, also capping and filling will be treated in a separate article. I am aware that there is much in the manner of preparing the gold and its introduction which is new, at least not so far as I know, practiced by the profession. I had never seen a pair of plugging forceps or plyers until I had them made. It is not expected that those accustomed to fill teeth in a different manner will (if even they see proper to try the use of the forceps with the foil rolled into blocks) succeed to their satisfaction the first effort—practice makes perfect in this as well as in almost everything. "Try all and adopt that which is best."

